# Visualizing set relationships: EVenn's comprehensive approach to Venn diagrams

**DOI:** 10.1002/imt2.184

**Published:** 2024-04-11

**Authors:** Mei Yang, Tong Chen, Yong‐Xin Liu, Luqi Huang

**Affiliations:** ^1^ Institute of Traditional Chinese Medicine Tianjin University of Traditional Chinese Medicine Tianjin China; ^2^ State Key Laboratory for Quality Ensurance and Sustainable Use of Dao‐di Herbs, National Resource Center for Chinese Materia Medica China Academy of Chinese Medical Sciences Beijing China; ^3^ Shenzhen Branch, Guangdong Laboratory of Lingnan Modern Agriculture, Genome Analysis Laboratory of the Ministry of Agriculture and Rural Affairs, Agricultural Genomics Institute at Shenzhen Chinese Academy of Agricultural Sciences Shenzhen China

**Keywords:** Euler diagram, flower plot, interactive Venn diagram, multiomics data visualization, standardized data formats, UpSet plot, Venn network diagram

## Abstract

Venn diagrams serve as invaluable tools for visualizing set relationships due to their ease of interpretation. Widely applied across diverse disciplines such as metabolomics, genomics, transcriptomics, and proteomics, their utility is undeniable. However, the operational complexity has been compounded by the absence of standardized data formats and the need to switch between various platforms for generating different Venn diagrams. To address these challenges, we introduce the EVenn platform, a versatile tool offering a unified interface for efficient data exploration and visualization of diverse Venn diagrams. EVenn (http://www.ehbio.com/test/venn) streamlines the data upload process with a standardized format, enhancing the capabilities for multimodule analysis. This comprehensive protocol outlines various applications of EVenn, featuring representative results of multiple Venn diagrams, data uploads in the centralized data center, and step‐by‐step case demonstrations. Through these functionalities, EVenn emerges as a valuable and user‐friendly tool for the in‐depth exploration of multiomics data.

## Highlights

- Comprehensive Venn functionality: EVenn introduces a unified platform with diverse Venn diagram tools, from interactive diagrams to network representations, catering to the intricate needs of multiomics data analysis across metabolomics, genomics, transcriptomics, and proteomics.
- Efficient data exploration: EVenn's data center streamlines exploration by supporting a standardized data format, allowing researchers to effortlessly upload and analyze data. The manuscript demonstrates its practical utility through representative results and detailed case demonstrations.
- User‐friendly interface: With a user‐friendly interface, EVenn simplifies the generation of various Venn diagrams, Euler diagrams, UpSet plots, and more. This protocol establishes EVenn as a valuable resource for researchers seeking a cohesive and accessible tool for multiomics data interpretation.

## Graphical abstract

The use of Venn diagrams greatly aids in illustrating and visualizing set relationships within metabolomics, genomics, transcriptomics, and proteomics. Here, we introduce EVenn, an all‐encompassing platform that provides a unified interface for a wide range of Venn diagram functionalities. This protocol delineates the features of EVenn, encompassing interactive Venn diagrams, interactive Edwards diagrams, Euler diagrams, UpSet plots, flower plots, and Venn network diagrams, solidifying its role as an invaluable tool for the analysis of multiomics data.

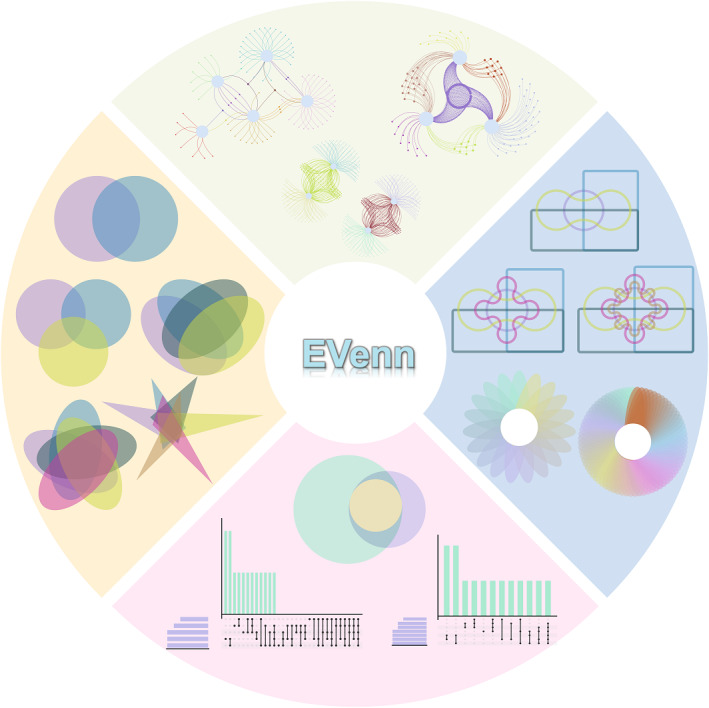

## INTRODUCTION

In recent years, with the rapid advancement of multiomics technologies, an increasing amount of data is being generated [1, 2]. Researchers are now faced with the challenge of processing and visualizing these vast amounts of multiomics data to gain meaningful insights into biological processes. The integration and visualization of intricate data sets have emerged as an increasingly crucial aspect of multiomics research [3, 4]. The data sets generated by these analyses are lists of differential metabolites [5], herbs [6], traditional Chinese syndromes [7, 8], comparison of differentially expressed genes [9, 10], the interaction between proteins and DNA/RNA [11, 12], microbiome specific [13, 14, 15, 16], and gene ontology (GO) similarity [17]. A commonly used approach to illustrate the intersections and distinctions among these datasets is through the utilization of a Venn diagram with tools like ImageGP [18], ComplexHeatmap [19], ggVennDiagram [20], Wekemo Bioincloud [21, 22].

Here, we present a comprehensive protocol showcasing the multiple Venn diagrams and diverse tool usage methods of EVenn [23]. The EVenn platform offers a range of features including data center, interactive Venn diagram, interactive Edwards diagram, high‐quality Euler diagram, UpSet plot, flower plot, interactive flower plot, Venn network, statistical significance computation for intersections, and Venn calculator for generating items and counts of all types of intersections for data with any number of sets. The functions are organized into separate tabs for efficient access, and each tab contains a demo data set to facilitate quick initiation. EVenn allows for the analysis and visualization across multiple data sets to explore and interpret complex multiomics data effectively. We present the applicability of each tool in EVenn for specific data set analyses, elucidate the meanings conveyed by various Venn diagrams, and outline the steps for data upload. Moreover, we demonstrate the versatility of EVenn through multiple use cases across different omics, highlighting its practicality in multidata analysis. The EVenn platform offers a valuable resource for researchers engaged in multiomics analysis, facilitating the exploration of novel insights and enabling data‐driven decision‐making across diverse biological contexts.

## INTERFACE AND REPRESENTATIVE RESULTS OF EVENN

The EVenn platform provides 10 modules on its main interface: data center, interactive Venn diagram, interactive Edwards layout, Euler diagram, UpSet plot, flower plot, interactive flower plot, Venn network, Venn estimate, and Venn calculator module. These modules including interactive Venn diagram, interactive Edwards layout, Euler diagram, UpSet plot, flower plot, and interactive flower plot can all be utilized for comparing differential metabolites of various tissues, gene expression profiles across different tissues or conditions, the protein composition in different samples, the presence of biomarkers across different diseases or conditions, screening results between different drug‐treated groups and control groups, and Operational Taxonomic Units (OTUs) in different samples [24]. The Venn network diagram is particularly valuable for the analysis of gene interactions, protein–protein interactions, and metabolite interaction networks. For instance, it can display overlapping genes and their associated gene Kyoto Encyclopedia of Genes and Genomes (KEGG) pathways or GO terms in a network diagram. This approach is also suitable for analyzing transcriptome, genome, and other omics data. In addition, these modules are used to compare data from different sets of quantities. Below, we provide detailed explanations that shed light on the representative result diagrams generated by each module.

### Interactive Venn diagram

**Figure 1:**
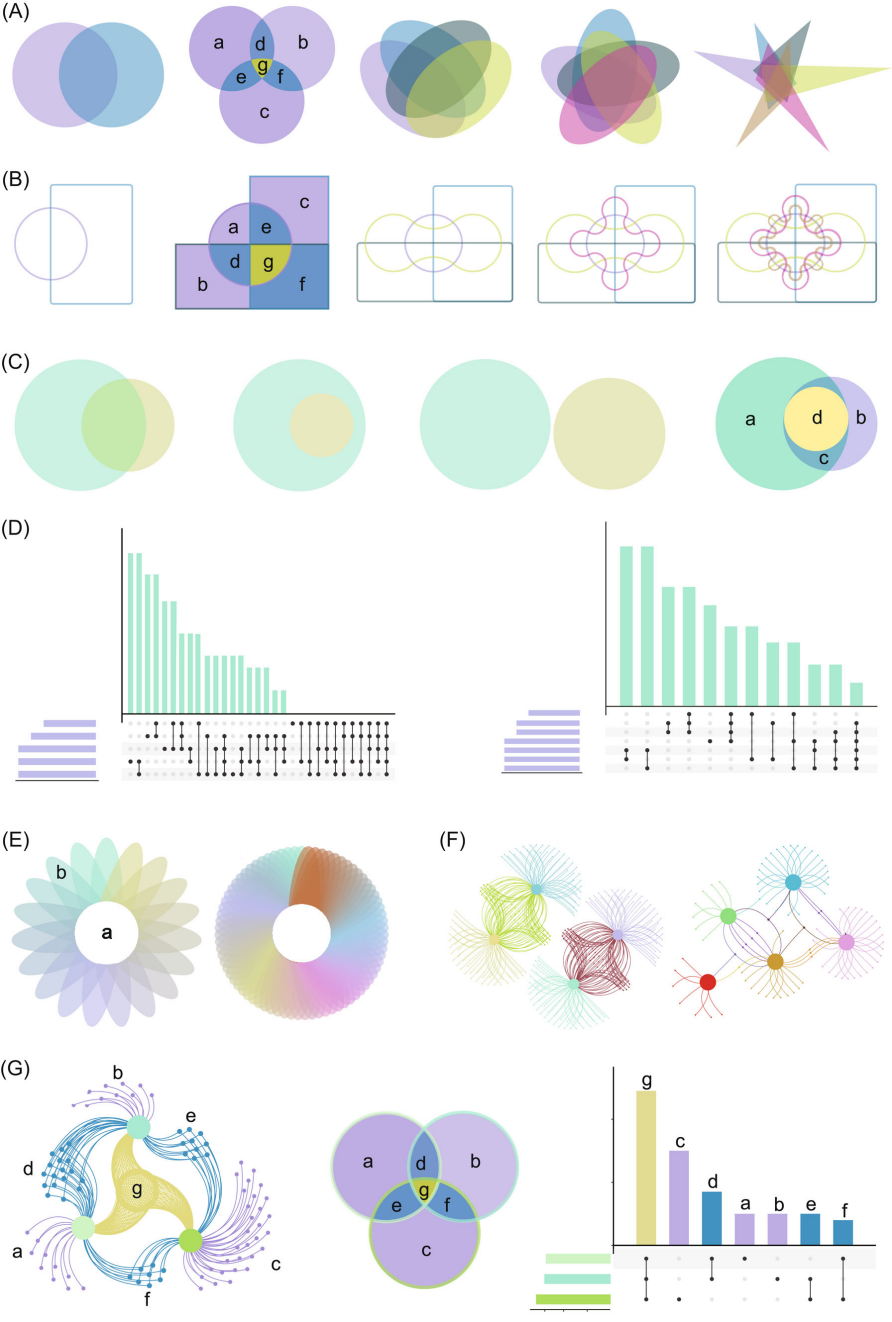
Representative result diagrams in EVenn. (A) Interactive Standard Venn diagrams for two to six sets. (B) Interactive Edwards diagrams for two to six sets. (C) Euler diagrams for two to three sets. (D) UpSet plot displaying empty intersections of five sets and nonempty intersections of seven sets. (E) Flower plot for 19 sets and 70 sets. (F) Venn network diagrams for four to five sets. (G) Interrelation among Venn diagram, UpSet plot, and Venn network.

The interactive Venn diagram within EVenn serves multiple purposes, allowing users to seamlessly switch between standard and Edwards Venn layouts while facilitating exploration and plotting for up to six sets. Standard Venn diagrams, commonly employed for comparing two to six sets of experimental data (Figure 1A), use color‐coded elements to represent unique and common components. For instance, purple (a, b, c) signifies elements unique to each set, blue (d, e, f) represents common elements between pairwise sets, and the yellow region (g) indicates the intersection of elements present in all three sets.

In contrast, Edwards Venn diagrams are introduced to enhance clarity in representing set intersections, particularly beneficial for cases involving five to six sets (Figure 1B). Using three sets as an example, where a, b, and c denote regions of unique elements, and d, e, and f represent regions of common elements in two sets, with g indicating common elements in all three sets. Edwards diagrams provide a more comprehensive visualization of overlapping elements, offering improved interpretability.

### Euler diagrams for enhanced visualization

Euler diagrams, in contrast to Venn diagrams, omit empty intersection regions whenever feasible, enhancing the precision and concreteness of visualization for multiple sets. Specifically designed for two and three sets of experimental data, Euler diagrams generate area‐proportional representations, where the sizes of intersection areas correlate with the number of shared elements. This feature allows for a clear depiction of partial‐containing, fully containing, and fully excluding relationships among sets (Figure 1C).

In the illustrated example with three sets in Figure 1C, each circle's size corresponds to the number of elements in its respective set, with larger circles indicating a greater number of elements. The distinct colors green (a) and purple (b) represent unique elements of each set, while the overlapping region (c) showcases shared elements between the two sets. Moreover, the yellow region (d) signifies the intersection where elements are shared by all three sets. These Euler diagrams effectively convey the inclusive relationships between sets, providing a nuanced visualization of set interactions.

### UpSet plot: A robust set intersection analysis

The UpSet plot emerges as an innovative visualization technique tailored for the quantitative analysis of set intersections, accommodating complex data sets with three to 40 sets. With two presentation modes, it adeptly visualizes both nonempty and empty intersections. Illustrated in Figure 1D, the plot comprises three key components: a horizontal bar plot portraying the total elements in each set, a vertical bar plot indicating elements in corresponding intersections, and a matrix with connected dots delineating all intersection types among sets.

In this example, individual points signify unique elements within each set, and connecting points symbolize shared elements between sets. The UpSet plot provides a comprehensive representation, with two connected points denoting elements shared by two sets, three connected points denoting elements shared by three sets, and four connected points indicating elements shared by four sets. This robust visualization tool facilitates nuanced analysis of intricate relationships within diverse data sets.

### Flower plots: Unveiling complexity in integrated data sets

When dealing with integrated data sets surpassing 10 sets, traditional visualization methods like Venn diagrams, Euler diagrams, and UpSet plots encounter limitations. Enter the flower plot—an invaluable tool for balancing interpretability and information richness. The left side of Figure 1E showcases a flower plot featuring 19 sets, while the right side unveils a variant with 70 elements rearranged.

In Figure 1E, the central circle (a) signifies elements shared across all sets. Surrounding it, (b) represents either the count of the unique elements in each set or the elements in each set post‐common element subtraction based on the parameter settings. The flower plot proves instrumental in unraveling intricate relationships within extensive data sets, offering a nuanced perspective beyond the capabilities of conventional visualization methods.

### Venn network: Unveiling interconnected set relationships

The Venn network transcends the conventional boundaries of standard Venn diagrams, moving beyond the mere depiction of intersections and unions. It artfully illustrates relationships within sets by designating each set as a parent node and connecting individual elements to their respective parent nodes via edges. The flexibility of Venn network layouts is demonstrated in Figure 1F, where diverse data types influence the resulting network structures.

In this illustration, network diagrams for four sets and five sets unveil the intricate relationships among their elements. Each element, connected by a single edge, remains distinct for its respective set. Two connected edges signify the intersection of two sets, while three edges represent the intersection of three sets. The Venn network provides a visual hierarchy of interconnectedness within the data sets.

### Harmony of Venn visualization trio: Venn diagrams, UpSet plot, and Venn network

In Figure 1G, we exemplify the synergy among Venn networks, Venn diagrams, and UpSet plots using a three‐set scenario. Each graph highlights element regions marked with corresponding letters, ensuring clarity in understanding relationships across the trio of visualizations. Consistency in labeling facilitates easy recognition of identical relationships, with shared letters indicating shared elements.

The vibrant color scheme aids in decoding relationships: yellow (g) denotes elements common to all three sets, while blue (d, e, f) signifies elements shared by pairs of sets. In contrast, purple (a, b, c) elegantly captures the distinctive elements unique to each individual set. This synchronized presentation offers a comprehensive understanding of set interrelations through a seamless integration of visual elements.

### Streamlined data input in EVenn's data center

The data center feature within EVenn streamlines the input process by supporting a standardized two‐column matrix format for interactive tools, including Venn diagrams, Euler diagrams, UpSet plots, flower plots, interactive flower plots, Venn networks, and the Venn calculator. This user‐friendly format, presented with the first row as the header line and the first column housing elements (e.g., genes or OTUs), ensures seamless compatibility across all tools. The second column corresponds to the respective sets, and a single TAB serves as the column separator.

Preparing this input matrix is a breeze, achievable through popular platforms like Excel, other compatible text editors, or simple programs. Users benefit from two convenient file upload modes offered by the data center: local file uploading and direct data pasting. This flexibility, combined with the standardized format, enhances user experience and accelerates the transition from data preparation to insightful analysis.

### Effortless file upload procedure

Uploading a file is a straightforward process. Begin by either dragging the file or clicking to initiate the upload (Figure S1, step 1). Assign a distinctive name to the file for easy identification (Figure S1, step 2). The uploaded file's content will be automatically populated into the designated text area (Figure S1, step 3), providing users with browsing and revision privileges as needed.

Ensure the completion of the uploading process by clicking the “Submit” button (Figure S1, step 4). Once done, your uploaded file will be prominently displayed in the designated file selector interface (Figure S1, step 5). This user‐friendly procedure ensures a seamless and efficient file upload experience.

### Efficient data pasting procedure

An alternative method for data input involves pasting your information directly into the data center. Begin by providing a file name for identification purposes (Figure S2, step 1), then seamlessly paste the data matrix into the designated text area (Figure S2, step 2). To complete the saving process, click “Submit” (Figure S2, step 3), and your uploaded file will be promptly displayed in the file selector (Figure S2, step 4).

To manage your uploads effectively, the data center allows a maximum of five files. To accommodate new additions, use the drop‐down menu below the “Submit” button to selectively delete older files before saving (Figure S2, step 5). It is crucial to note that these files are stored locally within your web browser and are not uploaded to our server. Consequently, closing your web browser will result in the loss of these uploaded files, and they cannot be accessed from other browsers.

### Intelligent limitations for data‐related parameters

To enhance user experience and prevent errors stemming from typing inaccuracies or misoperations, EVenn incorporates intelligent constraints for data‐related parameters. For instance, the Euler diagram accommodates two types of input matrices, with conditionally enabled/disabled settings for column information. In Two‐Column Mode, adjustments are limited to the Column containing all elements and the Column containing all sets of information. Moreover, a user‐friendly drop‐down box featuring predefined column names replaces an open‐ended input box, mitigating typos and offering valuable hints. These optimizations significantly contribute to the user‐friendly interface of EVenn, ensuring a smoother and more error‐free experience.

## CASE DEMONSTRATION

### Case I: An interactive Venn diagram illustrating differential metabolites

Venn diagrams offer a visually appealing approach to compare and analyze the overlap of metabolites, genes, and OTUs across multiple conditions or groups [25]. For instance, the differential metabolites of various parts of *Poria cocos* before and after specified treatment were investigated (Table S1). The interactive Venn diagrams offer support for three distinct methods of inputting data: uploading or pasting all data together using two‐column formats (default mode) (Figure S3, step 1), directly pasting elements of each set (input elements) (Figure S3, step 2), and manually entering counts of each intersection (input numbers) (Figure S3, step 3). Here, we outline a straightforward process utilizing a pasted two‐column matrix input approach to generate an interactive Venn diagram that effectively illustrates differential metabolites across five parts of *Poria cocos*. The method encompasses a series of steps outlined below:

1. *Data matrix input*: Paste the data matrix of differential metabolites in a two‐column format into the text area (Figure S4, step 1). The first column contains the metabolites and the second column contains the set information.
2. *Selection of set*: Select FS1, FS2, FS3, FS4, and FS5 sets in the specified order for analysis (Figure S4, step 2).
3. *Generating the interactive Venn diagram*: After completing the selections as mentioned above, an interactive Venn diagram is generated to display the intersections of differential metabolites within the five chosen parts (Figure S4, step 3).
4. *Rearranging set order*: Rearrange the order of the set (FS1, FS2, FS3, FS4, and FS5) by clicking and dragging them to the desired positions (Figure S4, step 4).
5. *Customizing set colors*: Customize the colors for the five sets using the color picker located next to each set name (Figure S4, step 5). Assign yellow, pink, green, purple, and blue colors to their respective sets.
6. *Global configuration parameters*: Activate the global configuration parameters by clicking on the designated button (Figure S4, step 6). Select “Classic mode” and “Edwards mode” accordingly. Keep default parameters for font family, font size, and statistics based on input lists, switch buttons, and intersection counts.
7. *Accessing specific intersection data*: Click on the count number within the Venn diagram to display differential metabolites associated with that intersection in the text area below (Figure S4, step 7). The headline indicates the affiliation of differential metabolites.
8. *Download options*: Obtain Venn diagrams of differential metabolites in PNG and scalable vector graphics (SVG) formats, as well as lists of intersection differential metabolites in CSV format, by clicking on the designated button (Figure S4, step 8).

**Figure 2:**
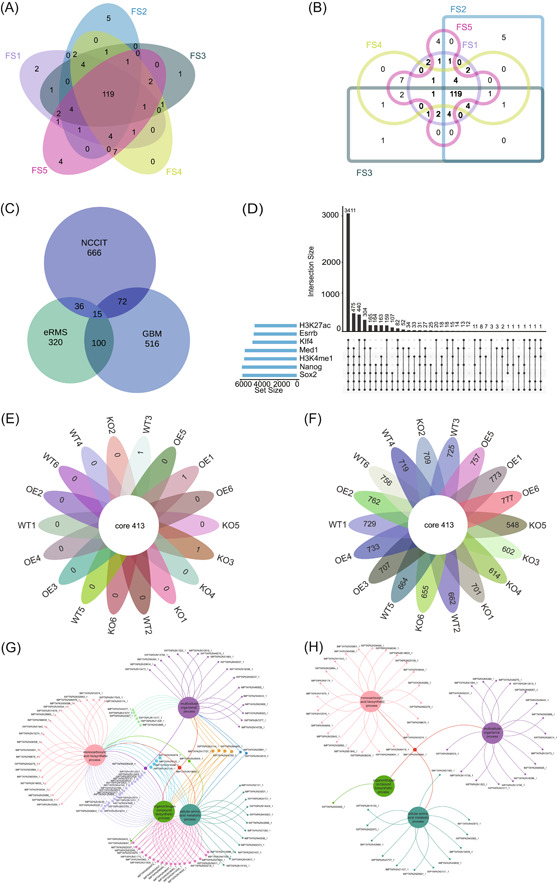
Case demonstrations in EVenn. (A) Interactive Venn diagrams for differential metabolites of five parts of *Poria cocos*. (B) Interactive Edwards diagrams for differential metabolites of five parts of *Poria cocos*. (C) Euler diagrams for differentially expressed genes in three cell types following anticancer drug treatment. (D) UpSet plot for seven transcription regulators and their targeting genes in ChIP‐seq. (E) Interactive flower plot displaying 18 groups of common and unique elements of OTUs. (F) Interactive flower plot showcasing OTUs of common and after subtracting common elements in 18 groups. (G) Venn network diagrams for visualizing gene ontology enrichment analysis results for *Andrographis paniculata*. (H) Venn network diagram exclusively showcasing common and unique genes of four biological processes of *Andrographis paniculata*.

The standard Venn diagram and Edwards diagram of differential metabolites in five parts of *Poria cocos* were depicted in Figure 2A,B, respectively. The collective analysis revealed a common pool of 119 identical differential metabolites across five distinct sets (FS1, FS2, FS3, FS4, and FS5). Among these sets, FS1, FS2, FS3, FS4, and FS5 displayed unique counts of 2, 5, 1, 0, and 4 differential metabolites, respectively. Further scrutiny uncovered interesting pairwise differentials: FS1 and FS2, as well as FS2 and FS3, exhibited 0 common metabolites; FS3 and FS4 shared 1, while FS4 and FS5 showed counts of 7, respectively. The Venn diagram clearly illustrated the distinct metabolic relationships between each part of *Poria cocos* compared to the other four parts.

### Case II: Euler diagram of differentially expressed (DE) genes

The utility of our Euler diagram tool is demonstrated by analyzing a published study that compares differentially regulated genes in different cell types following anticancer drug treatment [10]. Our Euler diagram tool accommodates two distinct input data methods: the two‐column mode format matrix and the count matrix, both comprising two columns. The specific steps for utilizing the tool to generate an Euler diagram of DE genes are as follows:

1. *Organizing data*: The number of DE genes for each cell type from the literature was organized and presented in Table S2.
2. *Selecting input type*: Choose the intersection‐count matrix method (Figure S5, step 1).
3. *Data input*: Paste the two‐columns count matrix of DE genes in the intersection‐count matrix (Figure S5, step 2). The first line should be treated as the header line. The first column contains intersection parts of all sets by rows, while the second column contains the number of elements in each intersection part. The “&” symbol in the first column represents intersecting. The column separator should be one TAB.
4. *Selecting essential parameters*: The parameter “Intersection column containing all intersections” is configured as “Intersection,” while the parameter “Column containing counts for each intersection” is set to “Count” (Figure S5, step 3).
5. *Additional style parameters*: Here, Layout and color and Picture attributes parameters utilize default settings (Figure S5, step 4).
  The complex color setting parameters are further explained. We provide two methods for defining colors: Manual color for each set and Manual color vector (color set) (Figure S6A). Manual color for each set: Set the color of each set. Please ensure to click on the “OK” button when selecting a color in the color pickers (Figure S6B). The usage of this parameter is mutual exclusion with the Manual color vector (color set) parameter. Please ensure that the values of the Manual color vector (color set) are cleared before enabling this parameter. If you want to assign a color for each set, the same number of colors should be picked (Figure S6B). If the number of picked colors is less than the number of sets, the program will generate intermediate colors to make them equal.
  Manual color vector (color set): Choose the color vector for all sets. This parameter is mutual exclusion with Manual color for each set parameter (Figure S6C). Please clear the values of the Manual color for each set parameter first to enable this parameter.
6. *Generating the Euler diagram*: Click on “Submit” (Figure S5, step 5) to display the Euler diagram of DE genes in the plot area (Figure S5, step 6). To save the image in PNG format, right‐click on it. Additionally, you can download the result picture in PDF format by clicking on the “Download PDF” button (Figure S5, step 7).

After meticulously following the outlined procedures, it is discernible that human embryonic carcinoma cells (NCCIT) demonstrated the highest count of unique genes, amounting to 666 and prominently occupying the largest area in Figure 2C. In contrast, the shared gene count between NCCIT, embryonic Rhabdomyosarcoma, and primary glioblastoma was minimal, totaling only 15 genes, thereby occupying the smallest area. Our tool excels in generating Euler diagrams with precise area proportions, thereby improving the accuracy of representing differential gene expression compared to existing publications.

### Case III: UpSet analysis of ChIP‐seq data

ChIP‐seq is a widely employed technique in genomics research for investigating protein–DNA interactions and comprehending the functional elements of the genome [26, 27]. In this case study, we conducted an analysis on ChIP‐seq data derived from five transcription factors (Esrrb, Klf4, Med1, Nanog, and Sox2) and two histone modification markers (H3K27ac and H3K4me1) in mouse embryonic stem cells (ESCs) to ascertain common and distinct binding sites. The ChIP‐seq data were obtained from the study conducted by Whyte et al. (Table S3) [28]. The UpSet plot accommodates two input formats: the two‐column mode format matrix and a binary matrix for illustrating the existence of elements. The step‐by‐step procedure for generating the ChIP‐seq UpSet plot is as follows:

1. *Preprocessing data set*: The transcription regulators Sox2, Nanog, Klf4, Esrrb, H3K27ac, Med1, H3K4me1 and their binding genes from Table S3 are selected utilizing the ImageGP platform (https://www.bic.ac.cn/BIC, Figure S7, step 1). The data are transformed into a two‐column format as shown in Table S4.
2. *Selecting input type*: The data is input using the selected two‐column format matrix.
3. *Data input*: The transcription regulators and targeting genes data should be pasted in a two‐column format, with the first column containing targeting genes and the second column containing information about the transcription regulators collection (Figure S8, step 1).
4. *Selecting essential parameters*: For the parameter “Treat the first line as header line,” it is set to “Yes,” while the parameter “Keep empty intersections” is set to “False” (Figure S8, step 2).
5. *Generating the UpSet plot*: The UpSet plot can be obtained by clicking the “Submit” button (Figure S8, step 3). The resulting image can be downloaded in PDF format by clicking the “Download PDF” button (Figure S8, step 4).

The UpSet plot illustrates the shared targets among the seven regulators as depicted in Figure 2D. A total of 3411 genes were found to be regulated by all seven transcription regulators (Sox2, Nanog, Klf4, Esrrb, H3K27ac, Med1, and H3K4me1). Furthermore, 475 genes exclusively bind to Sox2, Nanog, Klf4, Esrrb, Med1, and H3K4me1. Notably, these seven transcription regulators lack unique binding genes individually. The intricate interrelationships among their binding sites are effectively visualized through the UpSet plot.

### Case IV: Interactive flower plot for microbiome specificity

Exploring microbiome specificity is crucial for understanding ecological roles, diagnosing diseases, unraveling evolution, and exploring biotechnological potential [15, 29, 30, 31, 32, 33]. An interactive flower plot serves as a valuable tool to display both common shared and unique sets among more than 10 microbiome sets, offering an alternative method. OTU data from distinct groups—knock‐out (KO), wild‐type (WT), and overexpression (OE)—were obtained via EasyAmplicon (Table S5) [14]. To generate an interactive flower plot of the microbiome analysis, follow these steps.

1. *Preprocessing data set*: Employ the ImageGP platform (https://www.bic.ac.cn/BIC, Figure S7, step 1) to convert the data from Table S5 into a two‐column format (refer to Table S6 for the transformed data) [18].
2. *Two‐column mode data matrix input*: Paste the data of microbial OTU into the designated text area of the data box (Figure S9, step 1). The first column should contain elements (here OTUs or ASVs), while the second column should specify set names (here sample groups).
3. *Select parameters*: Choose the “OTUID” column for elements and the “Group” column for sets information (Figure S9, step 2). For the Ellipse numbers parameter, select two modes separately (Figure S9, step 3). All other parameters remain as default settings.
4. *Generating the interactive flower plot*: Click on “Submit” to generate the interactive flower plot (Figure S9, steps 4 and 5). Microbiome common and special elements can be obtained by clicking on the numbers in the image (Figure S9, step 6).
5. *Download SVG*: Export the resulting image in SVG format.

Figure 2E illustrates 413 identical OTU elements distributed across 18 sample groups. Among these, the sets WT3, OE1, and KO3 contain unique ASVs, namely ASV_942, ASV_1022, and ASV_1201, respectively. Conversely, the remaining 15 groups lack unique elements. In Figure 2F, the central circle reiterates the presence of 413 identical OTU elements across the 18 microbial groups. The 18 petals surrounding the circle represent the count of OTU elements in each group after subtracting common elements. Utilizing two different flower plot display methods proves advantageous for analyzing both similarities and differences in OTU within diverse microbial communities from multiple perspectives.

### Case V: Venn network showing GO enrichment results

To demonstrate the functionality of Venn network visualization, we obtained gene and GO data for *Andrographis paniculata* through the GO/KEGG enrichment analysis tool provided on the IMP (https://www.bic.ac.cn/IMP) platform [34]. The acquired data set, as depicted in Figure S10, encompasses biological process description and target genes. Our objective is to construct a Venn network diagram that highlights the interplay between genes and biological processes related to *Andrographis paniculata*. This involves a systematic approach:

1. *Preprocessing data set*: Select enriched GO enriched biological process terms, input them into the data conversion platform of the ImageGP (https://www.bic.ac.cn/BIC) platform, and convert the data into the two‐column patterns required by the Venn network (Figure S7, step 2, Table S7) [18].
2. *Data matrix input*: Paste the target genes in the first column and biological process description data in the second column to the text area (Figure S11, step 1).
3. *Selecting parameters*: Parameters are entirely optional, encompassing both color and mode. For illustrative purposes, we have chosen pink, green, purple, and blue as our color parameters (Figure S11, step 2). Additionally, the “Show all elements” mode was initially selected for comprehensive data representation, followed by the selection of the “show only common elements and specific elements like flower” mode for partial data display (Figure S11, step 3).
4. *Generating the Venn network diagram*: Upon clicking the “Submit” button, the Venn network diagram is generated (Figure S11, step 4).
5. *Optimize the layout of the Venn network diagram*: To optimize the clarity and efficacy of the Venn network diagram, access the “Tools menu” (Figure S11, step 5) and utilize the “Preferred layout” button (Figure S11, step 6). Subsequently, click on the button to unveil the left toolbar (Figure S11, step 7) for configuring additional parameters, encompassing nodes, edges, layout, and physics parameters. In this context, the edge type is preconfigured as “diagonalCross,” and the physics is preset to “barnesHut,” while other settings remain at their default values (Figure S11, steps 8 and 9).
6. *Edit X‐nodes and edge parameters for Venn network*: We supply an “Edit X‐nodes” button for altering the attributes of multiple selected nodes, and an “Edit Edge” button for changing the width and color of edges (Figure S11, steps 10 and 11). The color schemes for nodes and edges are illustrated in Figure 2G.
7. *Download Venn network diagram*: Click on the “Export SVG” button to export the Venn network diagram in SVG format (Figure S11, step 12).

As illustrated in Figure 2G, the IMPTAPA2N19692_1 gene is implicated in four crucial biological processes: monocarboxylic acid biosynthesis, cellular amino acid metabolism, organitogen compound biosynthesis, and multiple organismal enrichment process. The genes IMPTAPA2N21328_1, IMPTAPA2N31674_1, IMPTAPA2N40577_1, IMPTAPA2N11277_1, IMPTAPA2N33800_1, and IMPTAPA2N22367_1 are associated with the monocarboxylic acid biosynthetic and multicellular organismal process. Additionally, IMPTAPA2N33400_1 is enriched in the organonitrogen compound biosynthetic process. Figure 2H depicts a diagram illustrating the presence of shared and unique genes across four distinct biological processes. The Venn network diagram visually represents the relationship between the target genes and these biological processes.

## CONCLUSION

The complexity of Venn diagram data analysis arises from inconsistent data formats and the lack of collaborative data analysis platforms. This protocol provides a comprehensive overview of the functionalities and operations of various modules within the EVenn platform, including the data center, interactive Venn diagram, Euler diagram, UpSet plot, flower plot, interactive flower plot, and Venn network analysis.

The comparison of various biological entities such as metabolites, genes, genomic regions, and microbial taxa across different experimental conditions often requires the use of specialized visualization techniques. Among these are interactive Venn diagrams, Euler diagrams, UpSet plots, flower plots, and interactive flower plots. Interactive Venn diagrams are particularly useful for comparing up to six sets of data, allowing for visual identification of commonalities and differences. In contrast, Euler diagrams are more suited for comparing two to three sets, representing quantitative relationships within each set through area ratios. UpSet plots offer the flexibility to compare a wide range of sets, from three up to as many as 40, making them ideal for complex data sets. The flower plot, on the other hand, is valuable when assessing common and unique elements across more than 10 sets, providing a clear visual representation.

Furthermore, Venn network diagrams serve as effective tools for analyzing interaction networks among genes, proteins, and metabolites. They offer insights into the relationships between multiple sets of experiments, illustrating complex interaction patterns. In summary, the choice of visualization method depends on the specific nature of the data and the complexity of the relationships being studied. By understanding the capabilities of each technique, researchers can effectively interpret and communicate their findings.

Our goal is to empower users to effectively harness the diverse features and capabilities of EVenn. The versatility of EVenn is demonstrated through the generation of plots across multiple examples in various omics fields. We anticipate that EVenn will become a valuable tool for scientists in a wide range of tasks, providing significant insights for research.

## AUTHOR CONTRIBUTIONS

Mei Yang wrote the manuscript and drafted the figures. Tong Chen, Yong‐Xin Liu, and Luqi Huang supervised and funded this project, and revised the manuscript. All authors have read the final manuscript and approved it for publication.

## CONFLICT OF INTEREST STATEMENT

The authors declare no conflict of interest.

## Supporting information


**Figure S1:** Effortless file upload procedure.
**Figure S2:** Efficient data pasting procedure.
**Figure S3:** Three types of input ways (Input matrix in two‐column, Input items separately for each set, Input numbers for each intersection) for interactive Venn diagram.
**Figure S4:** Displaying the steps for generating interactive Venn diagram of differential metabolites with pasted data matrix in two‐column mode.
**Figure S5:** Displaying the steps for generating Euler diagram of DE genes with pasted data matrix in two‐column mode.
**Figure S6:** The way to set colors manually.
**Figure S7:** The BIC platform (https://www.bic.ac.cn/BIC) is employed for data conversion.
**Figure S8:** Displaying the steps for generating UpSet plot of ChIP‐seq with pasted data matrix in two‐column mode.
**Figure S9:** Displaying the steps for generating interactive flower plot of OTUs with pasted data matrix in two‐column mode.
**Figure S10:** The gene and GO data for *Andrographis paniculata* were obtained through the GO/KEGG enrichment analysis tool provided by the IMP platform (https://www.bic.ac.cn/IMP).
**Figure S11:** Displaying the steps for generating Venn network diagram of biological process description and target gene with pasted data matrix in two‐column mode.

**Table S1:** The differential metabolites of five parts of *Poria cocos*.
**Table S2:** The count matrix of differentially regulated genes of the three cell types after an anticancer drug treatment.
**Table S3:** Enhancers in ESCs, their locations, their associated genes, and background‐subtracted total ChIP‐seq reads per million values for Oct4, Sox2, Nanog, Med1, H3K27Ac, Klf4, and Esrrb.
**Table S4:** The ChIP‐seq data for Sox2, Nanog, Klf4, Esrrb, H3K27ac, Med1, H3K4me1.
**Table S5:** Abundance of OTUs in knock‐out (KO), wild‐type (WT), and overexpression (OE) microbiome.
**Table S6:** The OTUs in knock‐out (KO), wild‐type (WT), and overexpression (OE) microbiome in two‐column format.
**Table S7:** The target gene and its annotated biological process description.

## Data Availability

The data that support the findings of this study are openly available in iMeta at https://onlinelibrary.wiley.com/journal/2770596x. This paper does not generate any new data. The data and scripts used are saved in GitHub https://github.com/Tong-Chen/venn-doc. Supplementary materials (figures, tables, scripts, graphical abstract, slides, videos, Chinese translated version, and update materials) may be found in the online DOI or iMeta Science http://www.imeta.science/.
