# Supplementary material for: Visualizing set relationships: EVenn's comprehensive approach to Venn diagrams
Source: Imeta. 2024 Apr 11;3(3):e184. doi: 10.1002/imt2.184 (PMC11183158; doi:10.1002/imt2.184)
Supplement: Supplementary file 1 — Figure S1: Effortless file upload procedure. Figure S2: Efficient data pasting procedure. Figure S3: Three types of input ways (Input matrix in two‐column, Input items separately for each set, Input numbers for each intersection) for interactive Venn diagram. Figure S4: Displaying the steps for generating interactive Venn diagram of differential metabolites with pasted data matrix in two‐column mode. Figure S5: Displaying the steps for generating Euler diagram of DE genes with pasted data matrix in two‐column mode. Figure S6: The way to set colors manually. Figure S7: The BIC platform (https://www.bic.ac.cn/BIC) is employed for data conversion. Figure S8: Displaying the steps for generating UpSet plot of ChIP‐seq with pasted data matrix in two‐column mode. Figure S9: Displaying the steps for generating interactive flower plot of OTUs with pasted data matrix in two‐column mode. Figure S10: The gene and GO data for Andrographis paniculata were obtained through the GO/KEGG enrichment analysis tool provided by the IMP platform (https://www.bic.ac.cn/IMP). Figure S11: Displaying the steps for generating Venn network diagram of biological process description and target gene with pasted data matrix in two‐column mode. [file IMT2-3-e184-s001.docx]

**Supporting information to:**

**Visualizing set relationships: EVenn’s comprehensive approach to Venn diagrams**

**Running title:** Comprehensive Venn diagram visualization using EVenn

Mei Yang^1,2^, Tong Chen^2^*, Yong-Xin Liu^3^*, Luqi Huang^2^*

^1^Institute of Traditional Chinese Medicine, Tianjin University of Traditional Chinese Medicine, Tianjin 301617, China

^2^State Key Laboratory for Quality Ensurance and Sustainable Use of Dao-di Herbs, National Resource Center for Chinese Materia Medica, China Academy of Chinese Medical Sciences, Beijing 100000, China

^3^Shenzhen Branch, Guangdong Laboratory of Lingnan Modern Agriculture, Genome Analysis Laboratory of the Ministry of Agriculture and Rural Affairs, Agricultural Genomics Institute at Shenzhen, Chinese Academy of Agricultural Sciences, Shenzhen 518120, China.

*Correspondence: chent@nrc.ac.cn (Tong Chen), huangluqi01@126.com (Luqi Huang), and [liuyongxin@caas.cn](mailto:liuyongxin@caas.cn) (Yong‐Xin Liu)

**
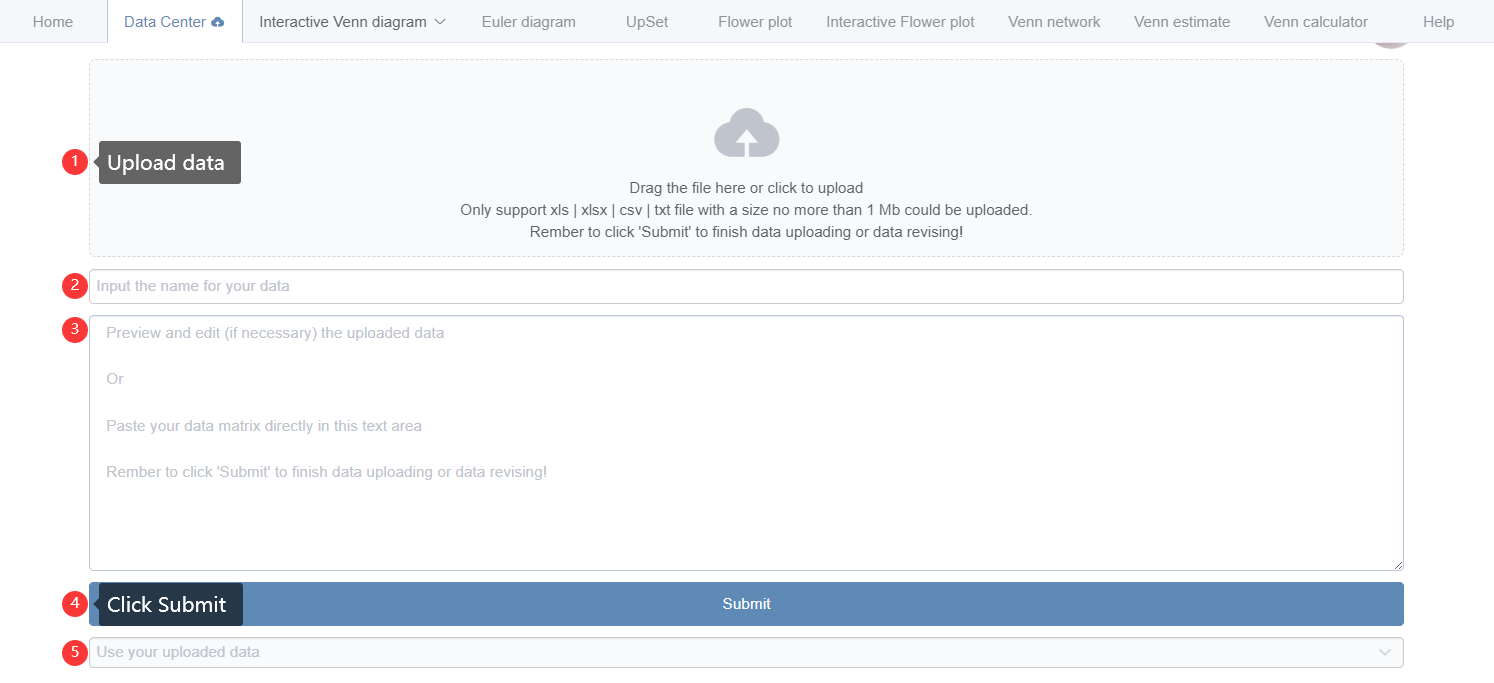
**

**Figure S1** **Effortless file upload procedure.** The data upload should be completed sequentially from step 1 to step 5 in a single operation.


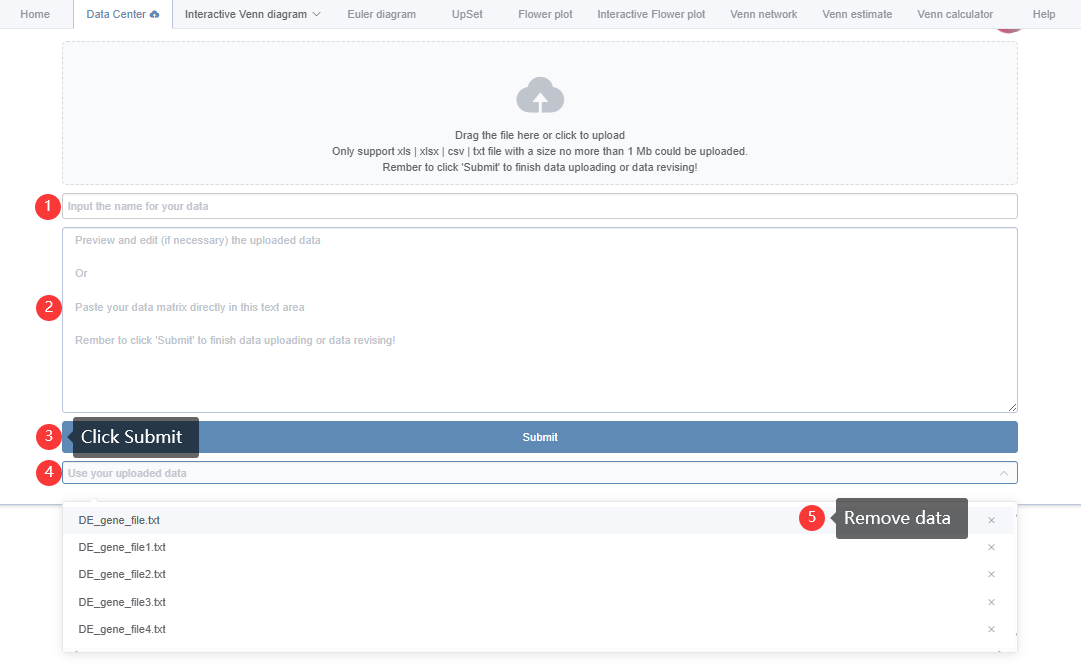


**Figure S2** **Efficient data pasting procedure.** The data paste should be completed sequentially from step 1 to step 5 in a single operation.


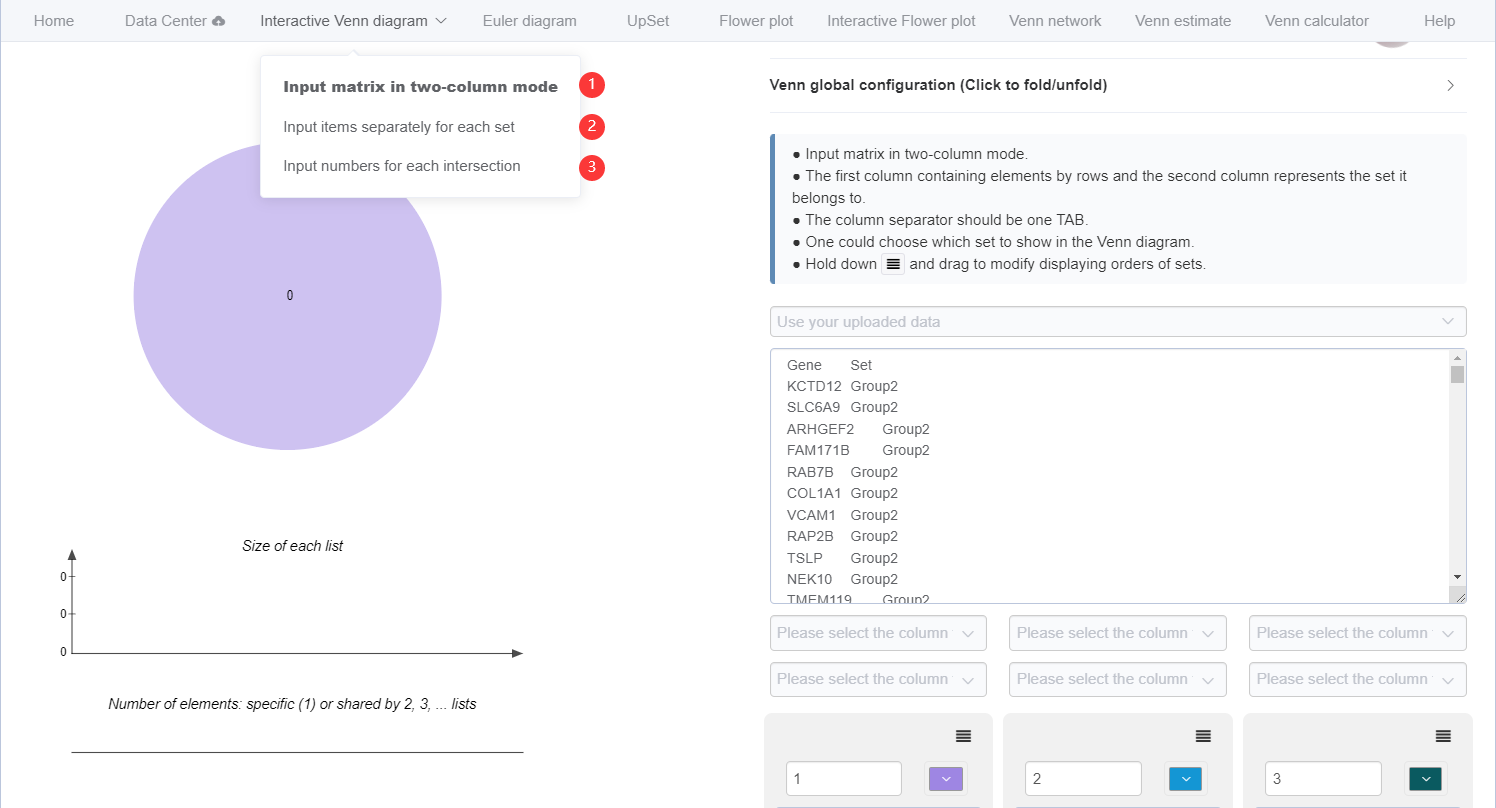


**Figure S3** **Three types of input ways (Input matrix in two-column, Input items separately for each set, Input numbers for each intersection) for interactive Venn diagram.**


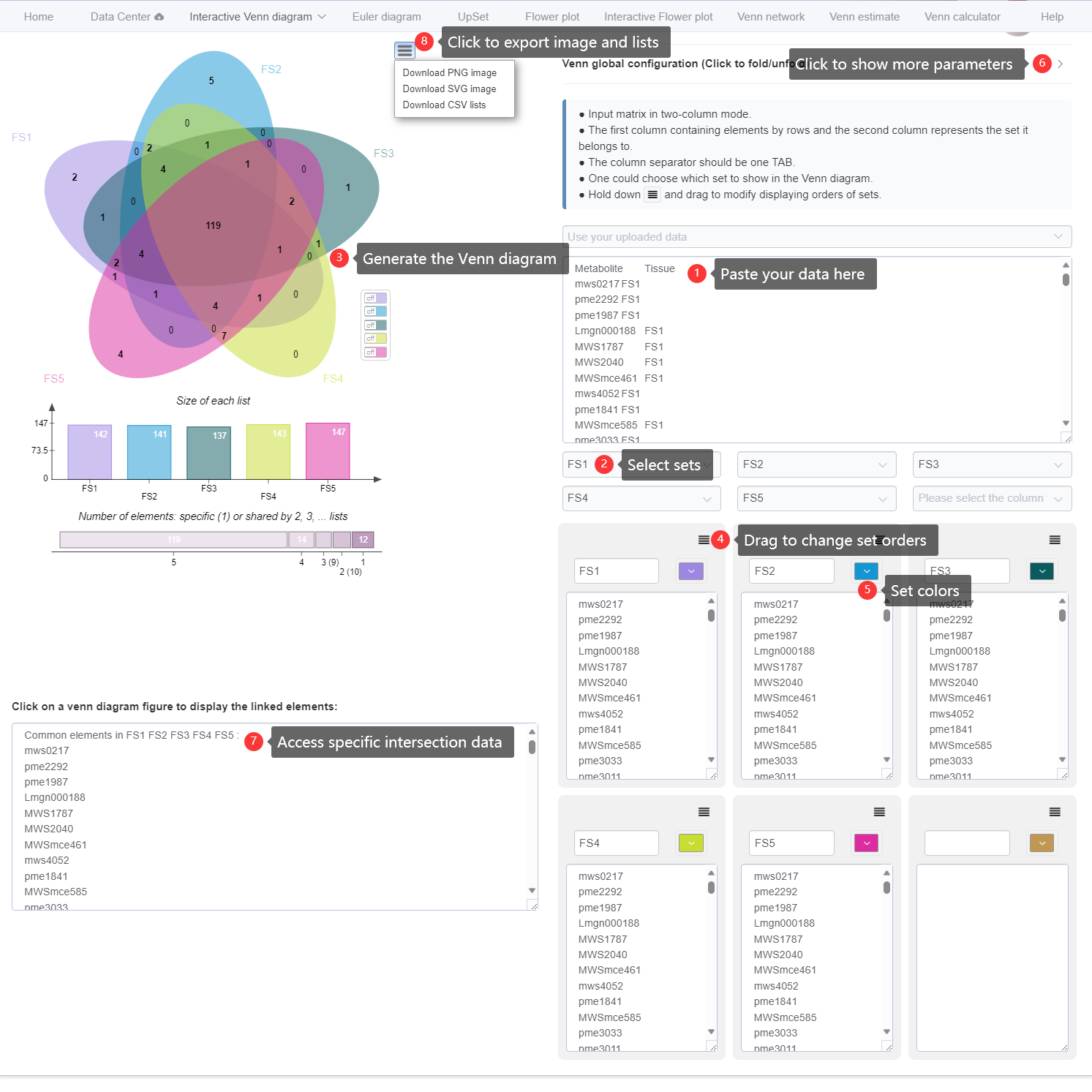


**Figure S4** **Displaying the steps for generating interactive Venn diagram of differential metabolites with pasted data matrix in two-column mode.**

**
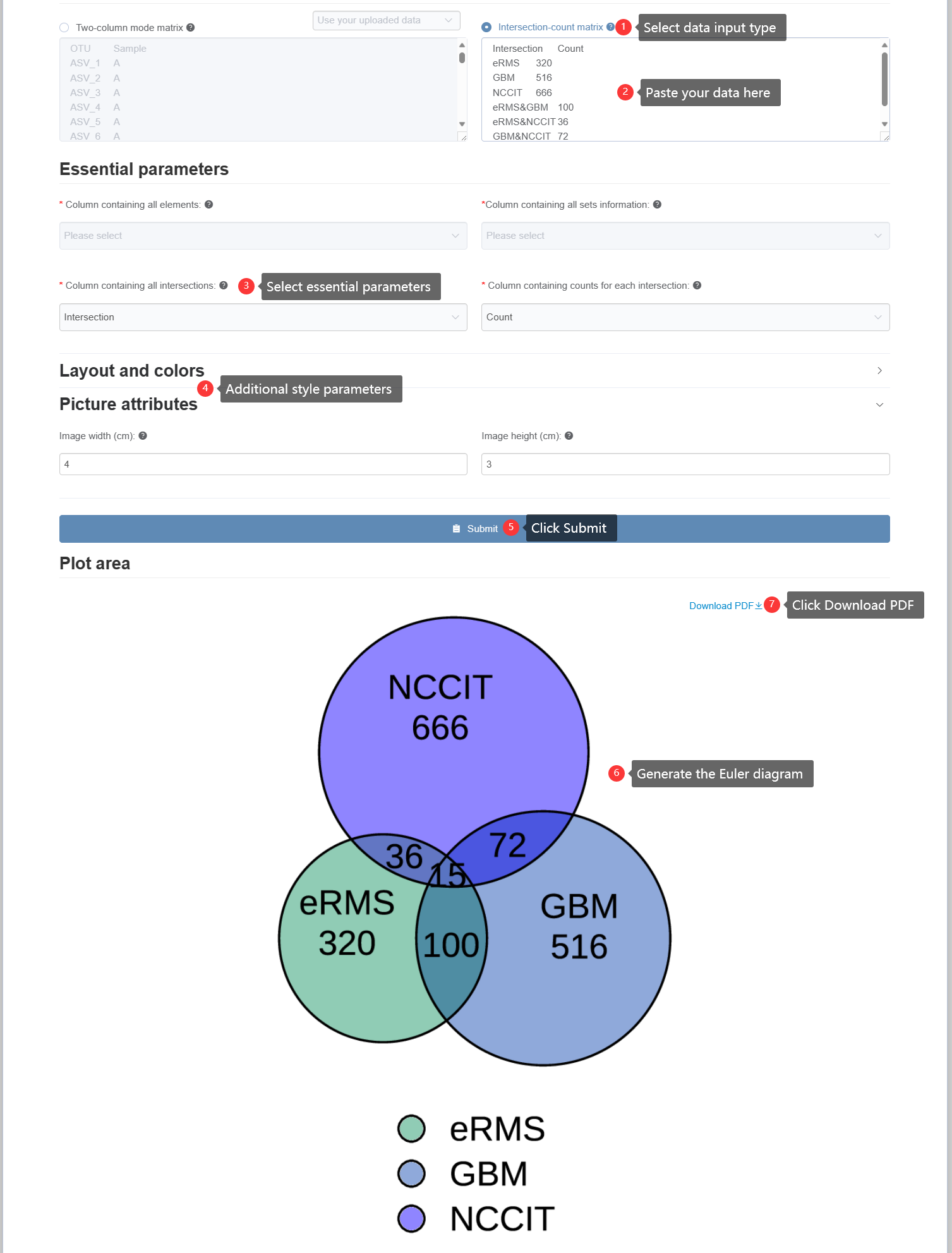
**

**Figure S5** **Displaying the steps for generating Euler diagram of DE genes with pasted data matrix in two-column mode.**


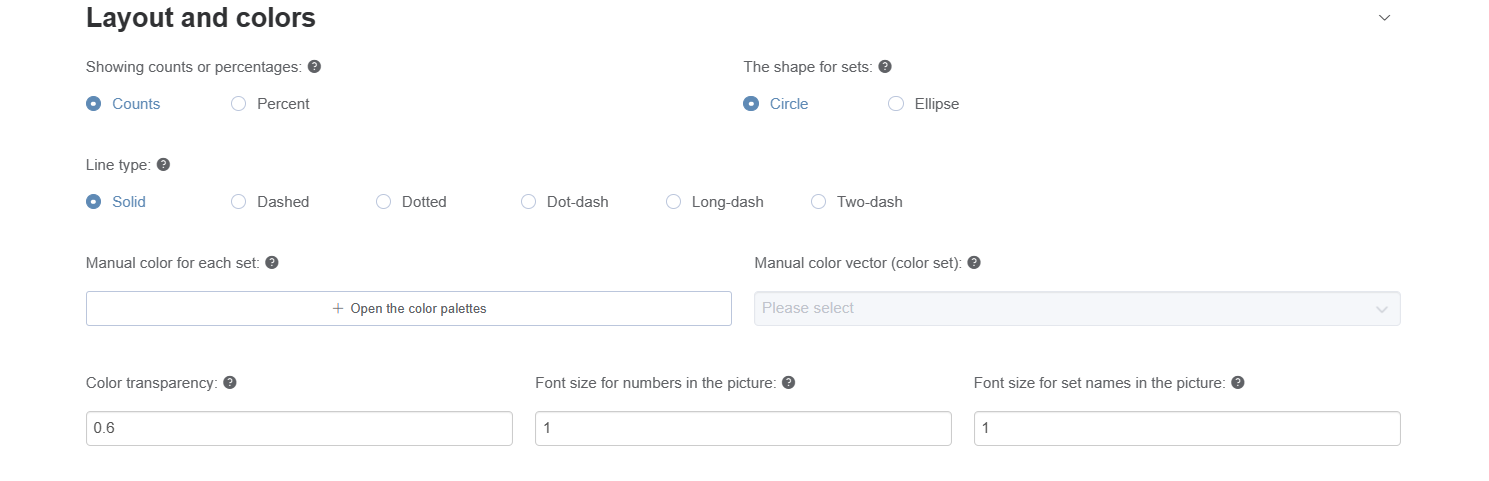


(B)

(A)


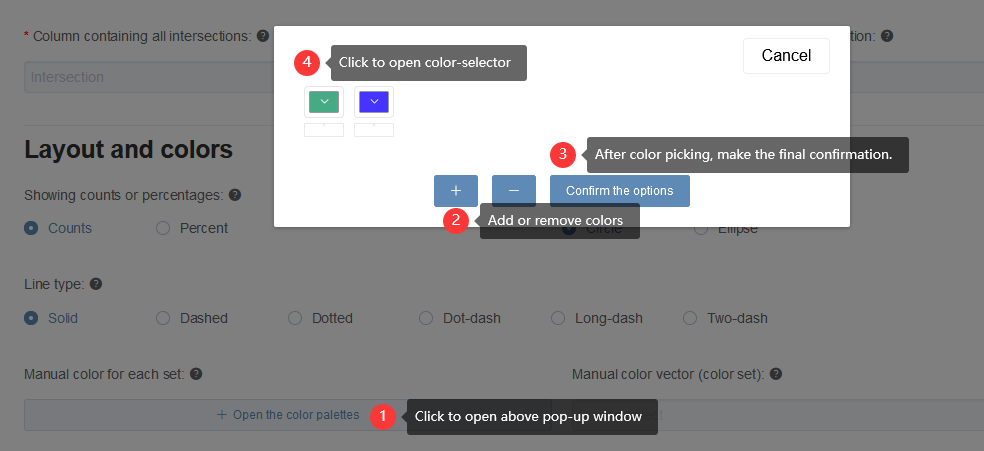


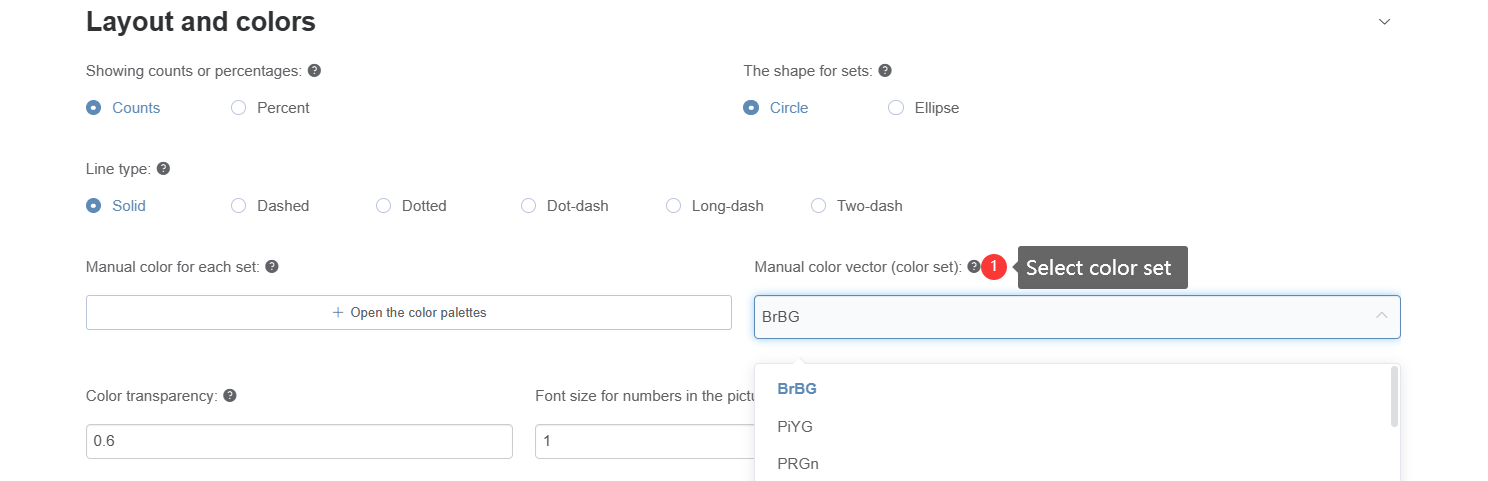


(C)

**Figure S6** **The way to set colors manually.** (A) Manual color for each set and Manual color vector (color set). (B) The way to set colors of Manual color for each set. (C) The way to set colors of Manual color vector (color set).


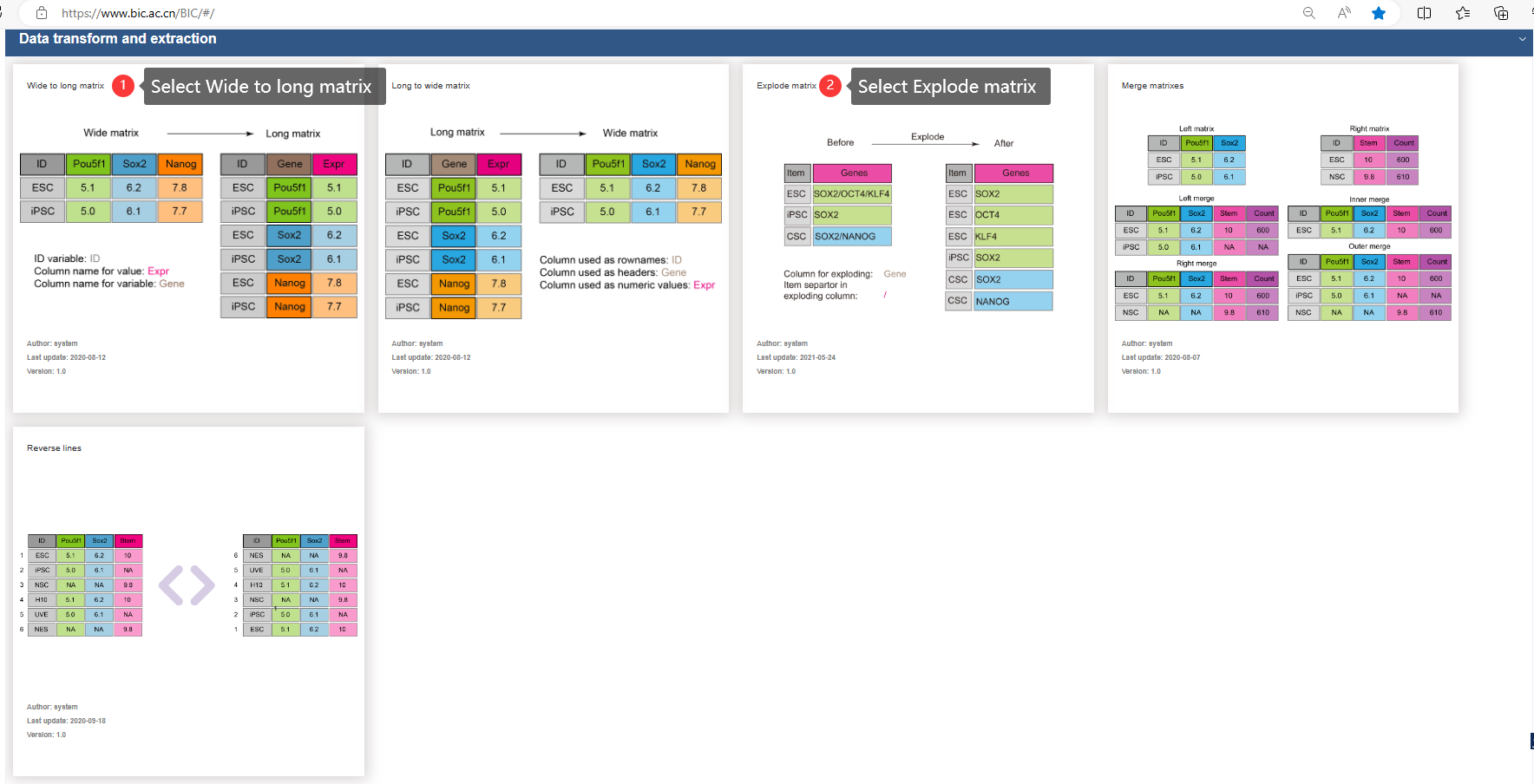


**Figure S7 The BIC platform (https://www.bic.ac.cn/BIC) is employed for data conversion.** The BIC platform is used in step 1 to convert wide matrix data into a two-column format. In step 2, the BIC platform is utilized to convert exploded matrix data into a two-column format.


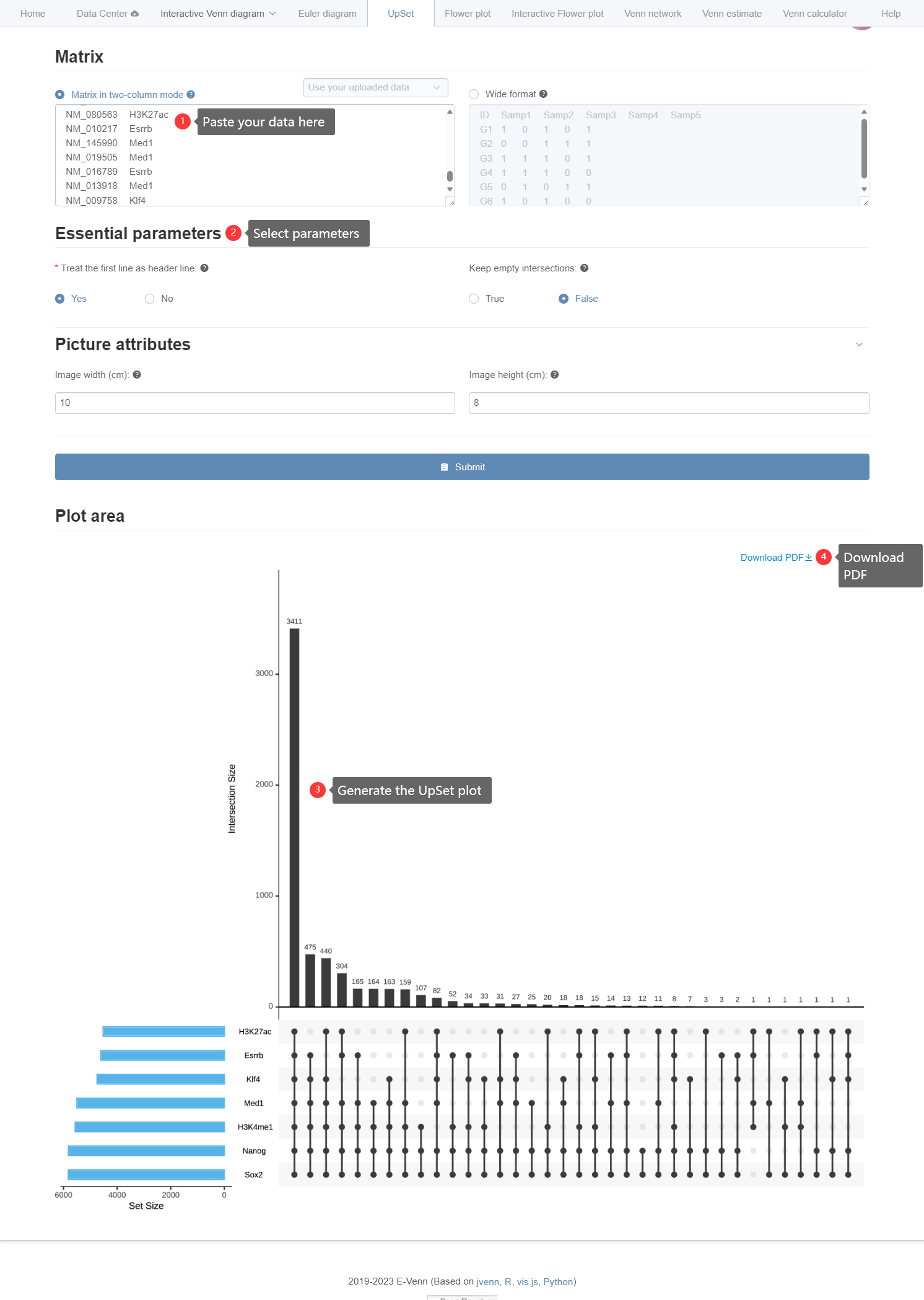


**Figure S8** **Displaying the steps for generating UpSet plot of ChIP-seq with pasted data matrix in two-column mode.**


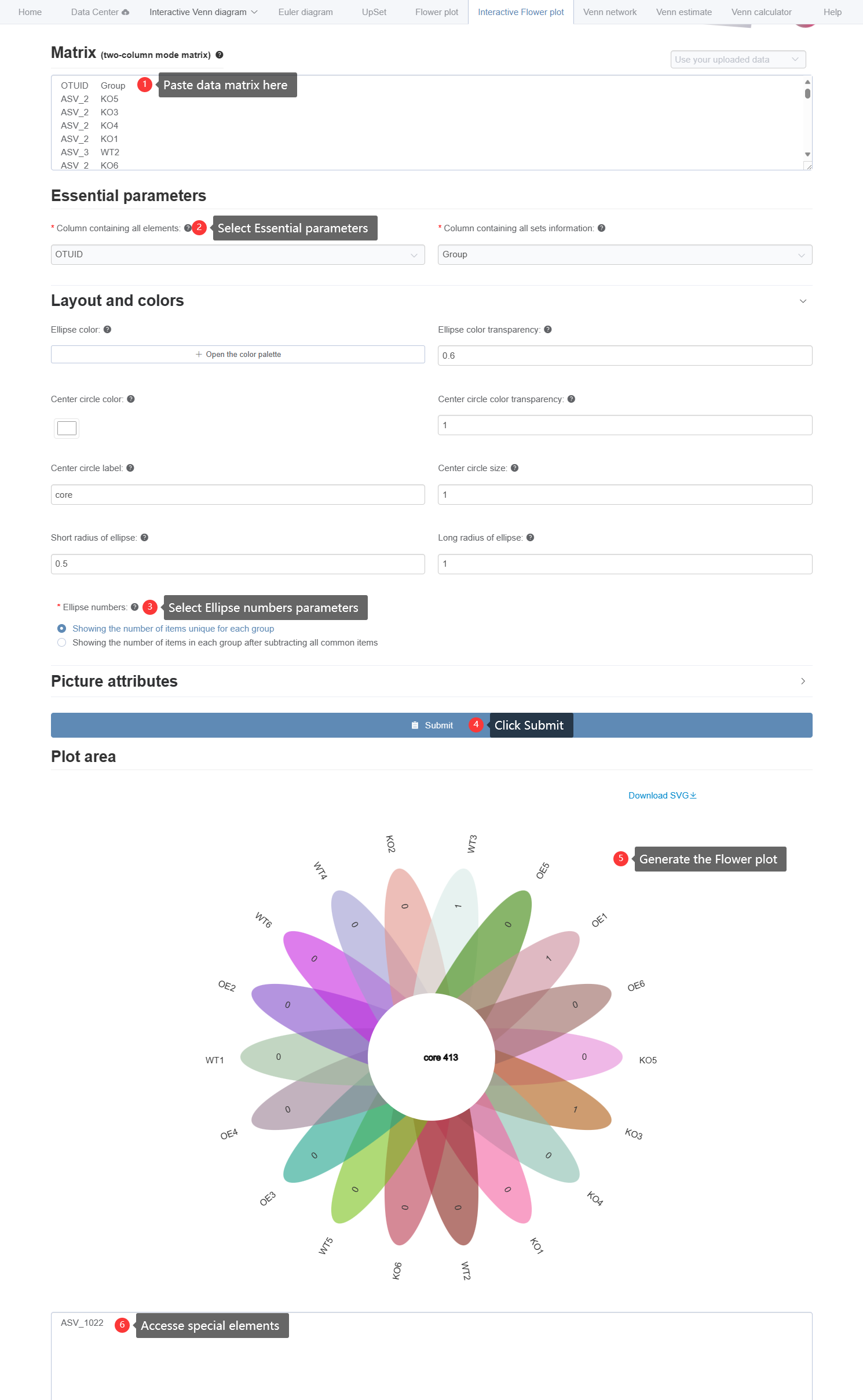


**Figure S9 Displaying the steps for generating interactive flower plot of OTUs with pasted data matrix in two-column mode.**


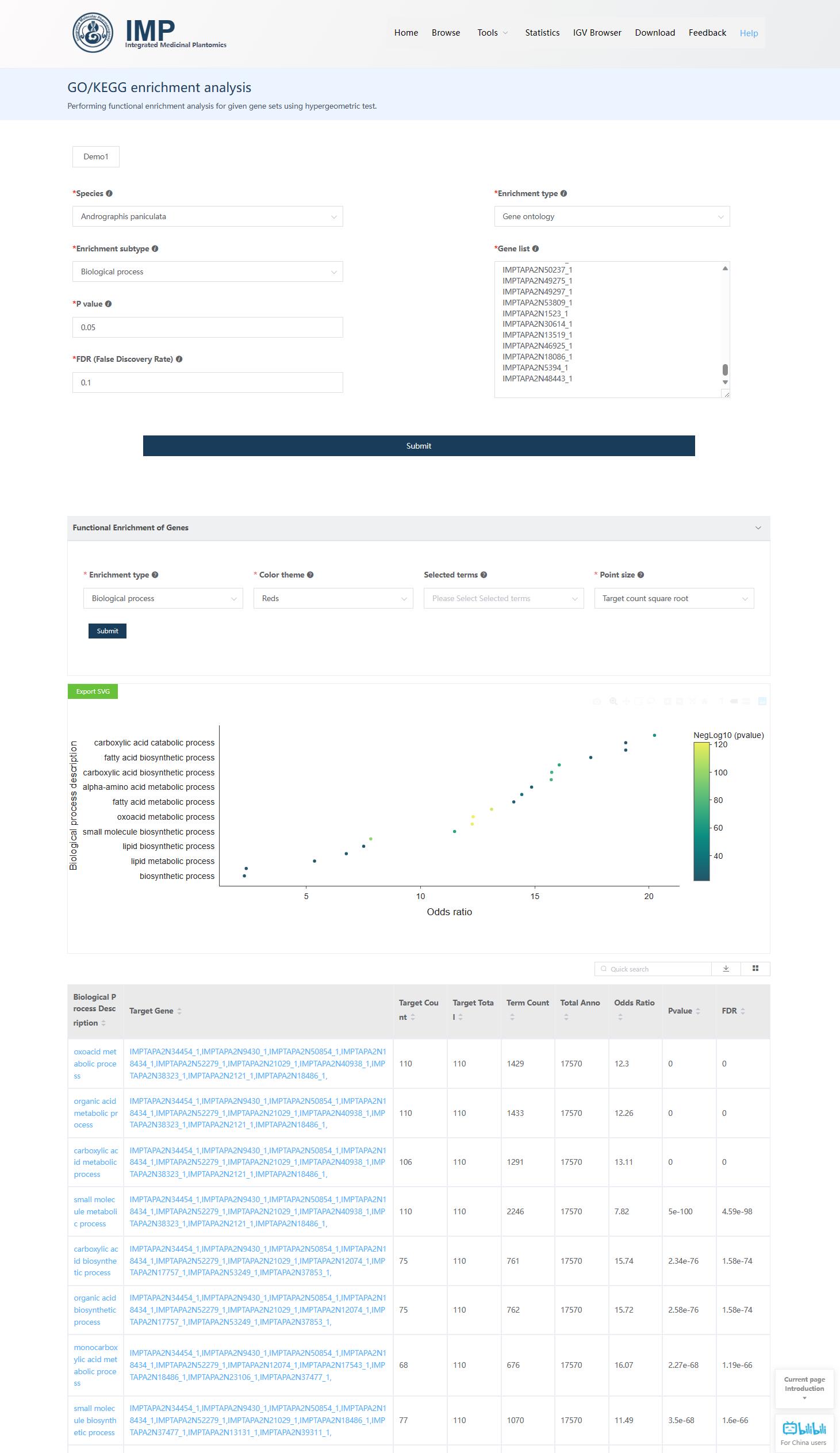


**Figure S10** **The gene and GO data for *Andrographis paniculata* were obtained through the GO/KEGG enrichment analysis tool provided by the IMP platform (**[**https://www.bic.ac.cn/IMP**](https://www.bic.ac.cn/IMP)**).**


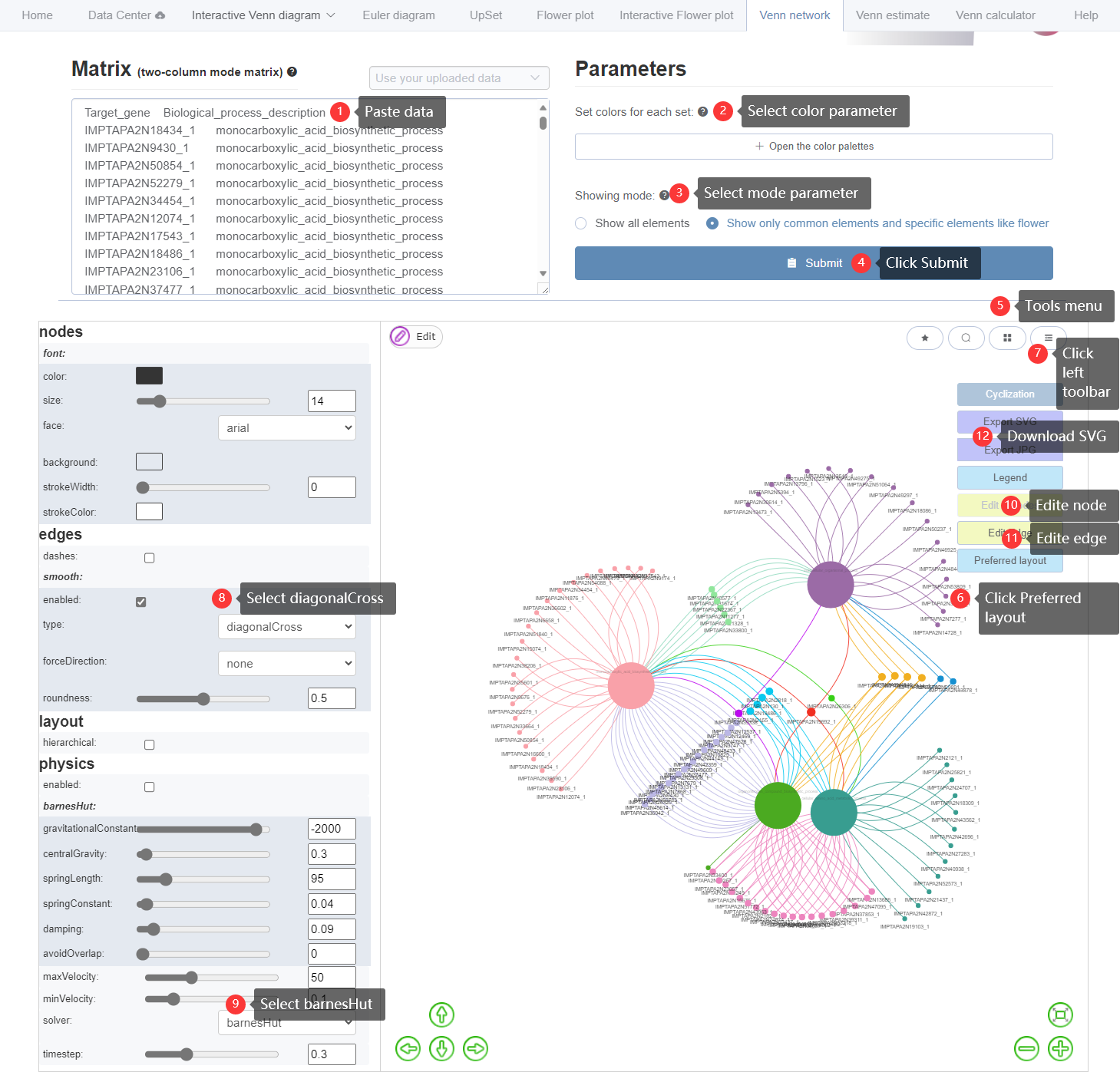


**Figure S11 Displaying the steps for generating Venn network diagram of biological process description and target gene with pasted data matrix in two-column mode.**
